# Adapting food environment frameworks to recognize a wild-cultivated continuum

**DOI:** 10.3389/fnut.2024.1343021

**Published:** 2024-04-09

**Authors:** Lilly Zeitler, Shauna Downs, Bronwen Powell

**Affiliations:** ^1^Department of Geography, The Pennsylvania State University, University Park, PA, United States; ^2^Department of Health Behavior, Society and Policy, Newark, NJ, United States; ^3^African Studies Program, The Pennsylvania State University, University Park, PA, United States

**Keywords:** natural food environment, diet quality, dietary diversity, indigenous, wild foods, niche construction theory, diet, Swidden

## Abstract

Food environments, or interfaces between consumers and their food systems, are a useful lens for assessing global dietary change. Growing inclusivity of nature-dependent societies in lower-and middle-income countries is driving recent developments in food environment frameworks. Downs et al. (2020) propose a food environment typology that includes: wild, cultivated, informal and formal market environments, where wild and cultivated are “natural food environments.” Drawing from transdisciplinary perspectives, this paper argues that wild and cultivated food environments are not dichotomous, but rather exist across diverse landscapes under varying levels of human management and alteration. The adapted typology is applied to a case study of Indigenous Pgaz K’Nyau food environments in San Din Daeng village, Thailand, using the Gallup Poll’s Thailand-adapted Diet Quality Questionnaire with additional food source questions. Wild-cultivated food environments, as classified by local participants, were the source of more food items than any other type of food environment (37% of reported food items). The case of Indigenous Pgaz K’Nyau food environments demonstrates the importance of understanding natural food environments along a continuum from wild to cultivated.

## 1 Introduction

Globalization is rapidly transforming diets and food choices (2, 3). An ongoing ‘nutrition transition’ toward calorie-dense and nutrient-poor ‘Western’ diets is exacerbating global burdens of disease (4), garnering much attention in the global public health literature. Changes to food environments are driving global dietary transitions (1). Often understood as the interface between the consumer and the food system (1, 5), food environments include physical environments where people acquire food (including built environments, such as homes, restaurants, schools, supermarkets; and natural environments, such as forests, home gardens and crop fields) with measurable characteristics that influence food decision-making (referred to as aspects or dimensions of the food environment, such as availability, access, affordability, convenience and desirability) (5–7).

Over the last few decades, food environment research has played a pivotal role in drawing attention to the structural factors shaping food access and choice (8, 9). Application of most food environment frameworks, however, remains limited to predominately high-income country and urban contexts with some notable exceptions (8, 9). The rest of the world (who still need to procure food and make dietary choices daily) are all-too-often overlooked. A recent systematic review of food environment research found no studies conducted in low-income countries (10). Since this systematic scoping review, a nascent food environment literature in low-income countries is emerging (11–15).

Geographic bias in the food environment literature toward high-income countries and urban contexts is responsible for an underrepresentation of some populations. The majority of the world’s Indigenous Peoples reside in low-and middle-income countries (LMICs). Indigenous communities are experiencing particularly stark agricultural and dietary transformations (16–18), associated with higher burdens of chronic disease (19). Indigenous food environments are shifting from wild and cultivated environments toward built food environments with reduced dependency on forest foods and increased market purchases (16, 20–22). Food environment frameworks designed for urban studies in high-income countries have not translated well to Indigenous and low-income country contexts, in which wild and cultivated landscapes and informal markets often provide important contributions to diet quality (1, 9, 15).

More inclusive frameworks for LMICs and Indigenous societies are beginning to garner attention (1, 12). Downs et al.’s typology of natural and built food environments is more inclusive of Indigenous and rural food environments, including those transitioning rapidly. Their framework fills a notable gap in the food environment literature for LMICs, where agricultural, pastoral, forested and aquatic natural environments provide affordable and healthy food sources in economically marginal contexts. Particularly commendable is the inclusion of wild foods. Though wild foods contribute substantially to the global food basket (23, 24), wild foods remain a key research gap in the food environment literature (5). Food environment research tends to underrepresent the contributions of wild foods and other non-market food sources in favor of the built market environment (6, 9).

New frameworks effective at drawing attention to the dietary significance of wild and cultivated natural food environments could further benefit from transdisciplinary perspectives. By leveraging findings from diverse disciplines, this paper reevaluates the wild-cultivated and nature-built dichotomies that permeate current food environment frameworks. Proposed is an adapted food environment typology that dissolves wild-cultivated boundaries in favor of a continuum. The adapted typology is applied to a case study of Indigenous Pgaz K’Nyau food environments in San Din Daeng village, Thailand, using the Gallup Poll’s Thailand-adapted Diet Quality Questionnaire (DQ-Q) (25). The Pgaz K’Nyau case study showcases the dietary importance of the previously overlooked wild-cultivated type of food environment in a semi-subsistence Indigenous community.

## 2 What is wild?

Current conceptualizations of the natural food environment designate ‘wild’ and ‘cultivated’ as separate spheres (1). Growing consensus in wild foods literature, however, contends that wild and cultivated environments vary along a ‘wild-cultivated continuum’ (23), by domestication stage (23), adaptive niches (26) and management intensity (27).

**Figure 1:**
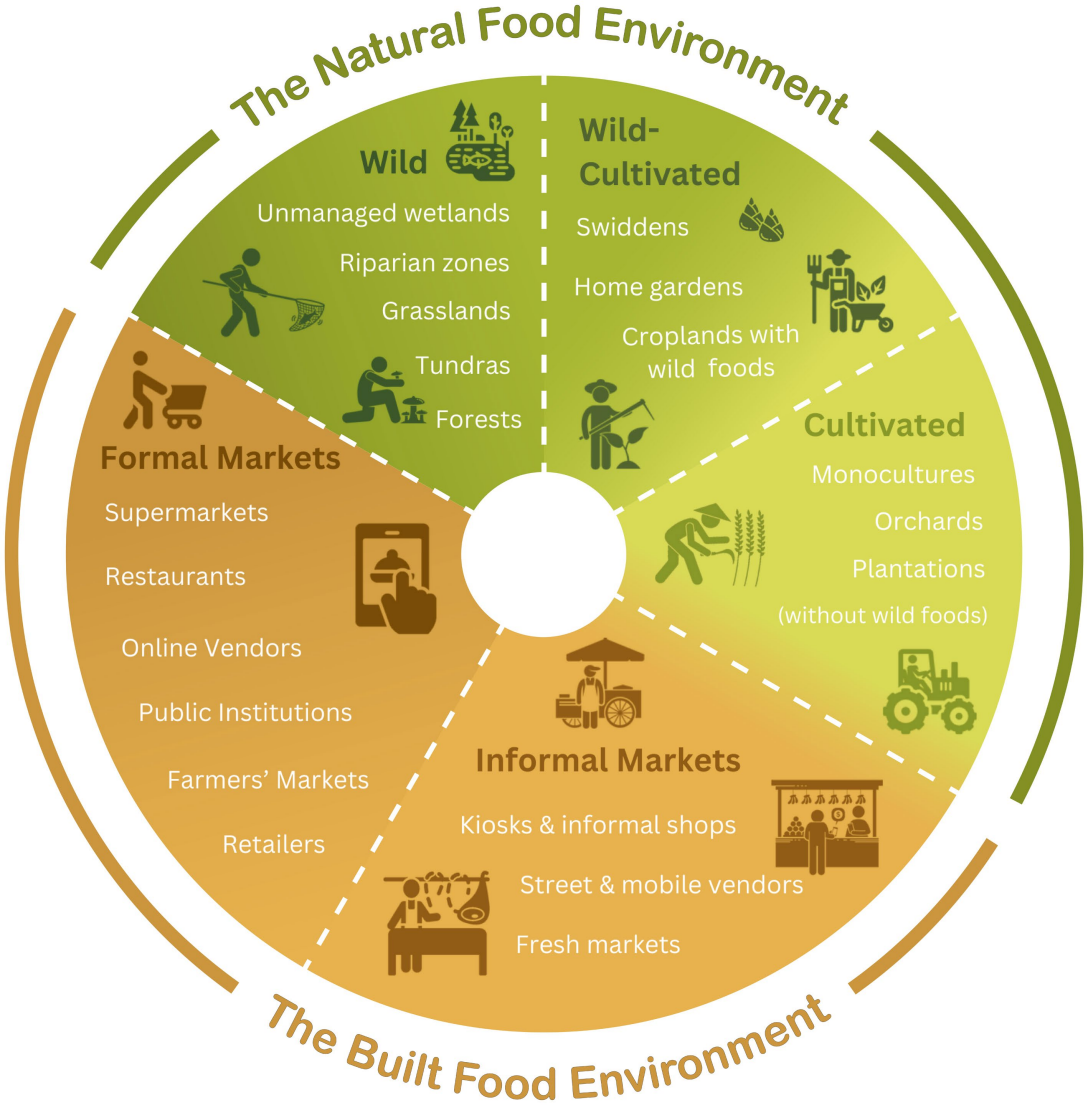
An adapted food environment typology with an integrated wild-cultivated continuum.

Wild-cultivated boundary-bending is the norm in many of the world’s natural food environments. Natural food environments, such as swidden fallows and home gardens, act as ‘boundary elements’ that traverse a wild-cultivated divide. Wild-cultivated crossovers include orchards or forests with lightly managed wild fruit trees (i.e., pruning, mulching and watering). Other wild-cultivated food environments include cultivation zones with wild terrestrial or aquatic foods, such as: (i) home gardens with wild transplants, spontaneous edible plants and bushmeat, (ii) rice paddies with wild plants, shellfish and amphibians, and (iii) swidden fallows with spontaneous vegetables and forest species (see Figure 1). Home gardens, for instance, have been described as the “closest mimics of natural forests yet attained,” signaling a status that is not purely cultivated nor fully wild (28). Swidden fields provide another liminal space traversing the wild-cultivated divide. Swidden forest-farmers modify landscapes with fire to create successional patch mosaics of croplands and secondary forests that provide ecological niches for a spectrum of wild-cultivated foods (29–31).

Niche construction theory (applied in human-environment geography, archeology, anthropology, ethnobotany, human ecology, among others fields) provides a theoretical explanation for the range of edible species’ adaptive niches that span a wild-cultivated continuum (26). Niche construction theory posits that originally ‘wild’ organisms adapt to environmental niches formed through human management and landscape modification. Commonalities in organism and landscape modification strategies derived from global case studies include: (i) modifying plant communities, (ii) broadcasting wild annuals, (iii) transplanting edible tree and root crops, (iv) light management of perennials (e.g., mulching and pruning), and (v) landscape modification for enhanced food procurement (32).

At the landscape-scale, following niche construction theory, alterations through fire or other disturbances, create niches for edible wild species to adapt. Human-landscape interactions, according to anthropologist, Paul Roscoe, complicate: (33).

“…what constitutes “wild.” The very presence of consuming humans on a landscape affects food resources, blurring the lines between wild and domesticated and, hence, between hunting and pastoralism and between gathering and cultivation (e.g., 34, 35).”

‘Natural’ landscapes, as noted by Roscoe, tend to be products of human modification. Iconic ‘wildernesses’, such as Yosemite Valley (36), the Amazon rain forest (37, 38), African savannahs (39) and Australia’s arid deserts (40, 41) have long-standing histories of anthropogenic manipulation for enhanced food acquisition. Forest-dwelling peoples around the world have long modified surrounding landscapes for hunting and foraging (30, 32). Artificial forest islands have transformed Southwest Amazonia (42) and African savannahs (39). Even remote jungles of the Amazon Basin rain forest are shaped by over 13,000 years of human-environment interactions (43), including shifting horticulture and tree planting since 4,000 years ago (~2,050 BC) (43), soil fertility enhancement (44–46) and ‘large-scale forest transformations’ (47). Amazonia and its jungles have even been referred to as a ‘domesticated landscape’ (48). Paleoethnobotanical explorations of ancient landscape management and agroecosystems have unearthed the co-existence of both wild and cultivated species in overlapping spaces (49). Findings from around the world demonstrate millennia of human-environment co-evolution that overturn wild-cultivated dichotomies (38, 42).

At the scale of organisms, classifying plants and animals as either wild or cultivated is similarly difficult, given the expanse of semi-cultivated states (23). Sago (*Metroxylon sagu* Rottboell), for instance, is situated along a management gradient from remote sago forest stands (with no or minimal management) to cultivated sago patches in villages (50). Sago users of Nuaulu ethnicity do not differentiate between wild and cultivated sago (50). Ethnobiologist, Roy Ellen, while working with the Nuaulu concluded that “there is a continuum,” because “the distinction between cultivated and non-cultivated becomes a difficult one to make” (50).

Scholars from diverse disciplines continue to grapple with the perplexing question of what ‘wild’ or ‘wilderness’ is (23, 50, 51). Food environment literature is beginning to engage with wild foods and wild natural environments (1), but not yet with the subtleties of bounding ‘wild’, ‘wildness’ and ‘wilderness’ that is highly contested in other fields.

## 3 An adapted food environment typology integrating a wild-cultivated continuum

The proposed conceptual approach leverages transdisciplinary perspectives on complex wild-cultivated dynamics to build upon Downs et al.’s natural (wild and cultivated) and built (formal and informal market) food environment typology to integrate a wild-cultivated continuum (1) (see Figure 1).

The adapted typology recognizes the oftentimes porous boundaries and complex crossovers and migrations of foods between different types of food environments (depicted with a dashed line in Figure 1). Some food items, such as fish, may be sourced from multiple different food environments, regardless of their original source. An artisanal fisherperson consuming their own wild-caught fish would be interacting with a wild natural food environment. Consumers purchasing wild-caught or aquaculture farmed fish in a supermarket would be interacting with a formal market environment.

We apply the adapted food environment typology to quantify the dietary contributions from different types of food environments in the Pgaz K’Nyau community of San Din Daeng village, Thailand.

## 4 Case study of indigenous Pgaz K’Nyau food environments in Thailand

Pgaz K’Nyau Peoples (a Karen ethnic subgroup) traditionally practice rotational farming, a type of shifting cultivation with 6–12 year fallows that support agrobiodiversity and dietary diversity (52). Forest conservation policies and market integration pressures are driving conversions toward monoculture, agrochemicals and market reliance. Simultaneously, highland infrastructure projects (e.g., roads, electricity) are increasing market access and altering local diets, resulting in Pgaz K’Nyau food environment transitions (1).

Dietary diversity from different food environment types was assessed in San Din Daeng village, Chiang Mai province, Thailand. Emic local classifications of types of food environments were discussed in focus groups (*n* = 6 women). Focus group participants classified food sources under the following types of food environments: (i) Cultivated: monoculture animal feed corn fields (indirect dietary pathway via income generation reinvested in market food purchases), (ii) Wild: forests (though forests are sites of animal husbandry, participants considered forests mostly ‘wild’), (iii) Wild-Cultivated: home gardens, swiddens, agricultural streams, and rice paddies (rice paddies were included due to the presence of aquatic wild foods), (iv) Informal Market: fresh markets, kiosks, street vendors, informal shops and restaurants, and (v) Formal Market: supermarkets (e.g., Tesco Lotus, Big C and Macro) and convenience stores (e.g., 7-11) located in Chom Thong town.

The Gallup Poll’s Thailand-adapted Diet Quality Questionnaire (DQ-Q) (25) was administered to one adult woman (>18 years old) per household (*n* = 31; 94% of households) in late rainy season (late September – October, 2023). Sources of food items consumed the previous day were also recorded (e.g., Cultivated: monoculture non-swidden crop field; Wild-Cultivated: home garden, swidden, rice paddy, agricultural pond or stream; Wild: forest or forest stream; Informal Market: fresh market, village kiosk, informal shop, informal restaurant, street vendor; and Formal Market: convenience store or supermarket) (see Supplementary Information for survey questions).

The average Dietary Diversity Score was 5.4 (ranging from 3 to 9) with 68% of respondents exceeding the Women’s Minimum Dietary Diversity Score of 5 (21 out of 31 women). Wild-cultivated environments were the most frequented type of food environments with respondents reporting daily visits on average (compared to 4 times per week for informal markets, once a month for wild environments, and less than once a month for cultivated and formal market environments).

**Figure 2:**
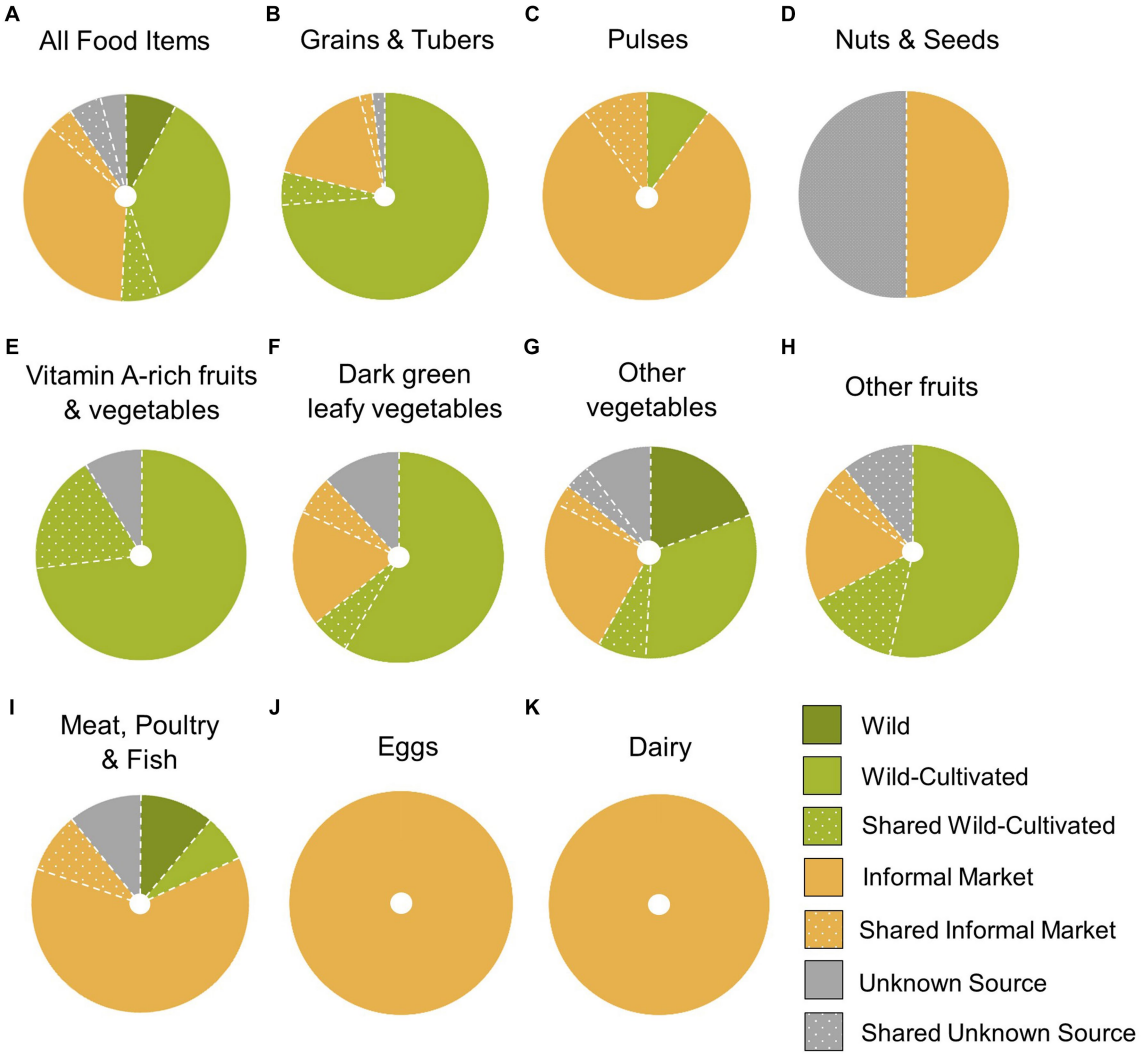
Proportions of food items acquired from each type of food environment per dietary diversity group **(A-K)**, reported in a diet quality questionnaire in San Din Daeng village, Thailand (*n* = 31; 240 food items). ‘Shared’ refers to food items acquired from food sharing. E.g. ‘Shared wild-cultivated’ refers to food items acquired via food sharing from a wild-cultivated food source.

More food items were consumed from wild-cultivated environments than any other type of food environment (37% or 88 out of 240 food items reported in the DQ-Q; see Figure 2). Wild-cultivated food environments were the main source of micronutrient-rich food groups (vitamin A-rich fruits and vegetables, dark green leafy vegetables, other vegetables and other fruit) consumed the previous day. The majority of vitamin-A rich fruits and vegetables were obtained from home garden and swidden wild-cultivated environments (91%, or 8 out of 11 food items with an additional 2 shared food items). Wild-cultivated environments provided 65% of dark green leafy vegetables (10 out of 17 reported food items, and 1 shared food item), 68% of other fruits (15 out of 28 food items with an additional 4 shared items), and 39% of other vegetables (21 out of 67 food items with an additional 5 shared items) (see Supplementary Table S1 in Supplementary Information). Animal-sourced foods, such as meat, fish and eggs, were predominately obtained from informal markets. Carbohydrate staples, such as rice, were mostly acquired from wild-cultivated swiddens and rice paddies (31 out of 37 reported grain food items, or 84%).

Despite rapid social-ecological change, San Din Daeng residents continue to rely heavily on natural food environments and particularly wild-cultivated environments. The formal market environment that has dominated food environment research is only marginal in this semi-subsistence setting (none of the food items reported in the DQ-Q were acquired from formal markets). The case of the Pgaz K’Nyau food environment of San Din Daeng village demonstrates that the previously overlooked wild-cultivated food environment can contribute substantially to local diets.

## 5 Discussion

Most food environment frameworks have underrepresented marginalized communities in LMICs, for whom the natural food environment presents a vital, affordable and healthy food source (1, 5, 53). With growing evidence on the nutritional importance of wild foods (54–57), the significance of natural food environments in LMICs is becoming more apparent (1, 5, 53). Though research on natural food environments is still embryonic, other disciplines have long engaged with forest-and nature-dependent peoples. Anthropologists, ethnobiologists, geographers, landscape ecologists, among others, have compiled an extensive body of knowledge on diverse food acquisition strategies, globally (41, 50, 55, 58). Greater emphasis on cross-disciplinary discussions is bringing decades of debates on wild-cultivated dynamics, plant-people interactions, traditional ecological and Indigenous knowledge, and nature-based ontologies into conversation with budding conceptual developments of natural food environments. Capitalizing on transdisciplinary theories, perspectives and bodies of knowledge can catalyze the development of effective food environment measurement tools that best capture the nuanced complexity of natural food environments.

## Data availability statement

The datasets presented in this article are not readily available because data is only made available upon request at the authors’ discretion. Requests to access the datasets should be directed to lmz5288@psu.edu.

## Ethics statement

The studies involving humans were approved by the Pennsylvania State University Institutional Review Board (STUDY00019694). The studies were conducted in accordance with the local legislation and institutional requirements. Written informed consent for participation was not required from the participants or the participants’ legal guardians/next of kin because some participants can not read or write. Participants signed or checked their names on a consent form. Formal written consent was obtained from the village leader.

## Author contributions

LZ: Conceptualization, Data curation, Formal analysis, Funding acquisition, Investigation, Methodology, Project administration, Resources, Visualization, Writing – original draft, Writing – review & editing. SD: Methodology, Supervision, Visualization, Writing – review & editing. BP: Conceptualization, Funding acquisition, Methodology, Project administration, Resources, Supervision, Visualization, Writing – review & editing.
