# Supplementary material for: Adapting food environment frameworks to recognize a wild-cultivated continuum
Source: Front Nutr. 2024 Apr 9;11:1343021. doi: 10.3389/fnut.2024.1343021 (PMC11035871; doi:10.3389/fnut.2024.1343021)
Supplement: Supplementary file 1 [file Data_Sheet_1_1.pdf]

# Supplementary Information

## The Gallup Poll’s Thailand-Adapted Diet Quality-Questionnaire with additional food environment questions in English and Thai language

| **เมื่อวานคุณได้รับประทานอาหารเหล่านี้หรือไม่**<br>Yesterday, did you eat any of the following foods: | |
|---|---|
| D1. **ข้าว, ข้าวเหนียว, ข้าวต้ม, ก๋วยเตี๋ยว, หรือขนมปัง**<br>Rice, sticky rice, porridge, noodles, or bread? | 0 = ไม่ใช่ 1 = ใช่ |
| D2. ข้าวกล้อง, ข้าวสีนิล, ข้าวโพด, ลูกเดือย, หรือ ข้าวโอ๊ต<br>Brown rice, black rice, corn, job’s tears, or oats? | 0 = ไม่ใช่ 1 = ใช่ |

| **D3. ข้าว และ ข้าวโพด ที่คุณได้กินเมื่อวาน ได้มาจาก ไหน**<br>From which of the following types of places did you get the rice and oats that you ate yesterday? | |
|---|---|
| 0 = **ไม่ได้กิน** Did not eat.<br>2= **ตลาดสด** Fresh market<br>2.5= **ร้านขายของชำ** Informal shop<br>3 = **สวนผลไม้** Fruit orchard<br>5 = **สวนรอบบ้าน** Home garden<br>6 = **นาข้าว** Rice paddy<br>8 = **ไร่หมุนเวียน ที่กำลังปลูกอยู่**<br>Planted rotational cropfield<br>10 = **ป่า** Forest | 1= **ห้างสรรพสินค้า หรือร้านสะดวกซื้อ เช่น เทสโก้ โลตัส หรือเซเว่น**<br>Formal markets like Tesco Lotus or 7- Eleven<br>4 = **สวนทำการเกษตรเชิงพาณิชย์** Cash cropfield<br>7 = **ไร่ / สวน** Subsistence crop field<br>9 = **ไร่หมุนเวียน ที่วางไว้** Fallow rotational field<br>11= **ริมลำห้วยที่ผ่านไปการเกษตร** Agricultural streams<br>12 = **ริมลำห้วย หรือแม่น้ำในป่า** Rivers and streams in forest<br>13 = **ริม ถนน ทางเดิน หรือรั้ว** Roadside<br>14 = **อาหารที่แบ่งปันกัน ระบุ** Shared food, specify:<br>15 = **ที่อื่น ระบุ** Other place, specify: |

| | |
|---|---|
| D4. **เมื่อวานคุณได้รับประทานอาหารเหล่านี้หรือไม่** มันสำปะหลัง, มันหวาน, มันฝรั่ง, เผือก, หรือมันแกว Yesterday, did you eat any of the following foods: Cassava, sweet potato, potato, taro or yam? | 0 = ไม่ใช่<br>1 = ใช่ |

| **D5. มันที่คุณได้กินเมื่อวาน ได้มาจาก ไหน**<br>From which of the following types of places did you get the potatoes and tubers that you ate yesterday? | |
|---|---|
| 0 = **ไม่ได้กิน** Did not eat.<br>2= **ตลาดสด** Fresh market<br>2.5= **ร้านขายของชำ** Informal shop<br>3 = **สวนผลไม้** Fruit orchard<br>5 = **สวนรอบบ้าน** Home garden<br>6 = **นาข้าว** Rice paddy<br>8 = **ไร่หมุนเวียน ที่กำลังปลูกอยู่**<br>Planted rotational cropfield<br>10 = **ป่า** Forest | 1= **ห้างสรรพสินค้า หรือร้านสะดวกซื้อ เช่น เทสโก้ โลตัส หรือเซเว่น**<br>Formal markets like Tesco Lotus or 7- Eleven<br>4 = **สวนทำการเกษตรเชิงพาณิชย์** Cash cropfield<br>7 = **ไร่ / สวน** Subsistence crop field<br>9 = **ไร่หมุนเวียน ที่วางไว้** Fallow rotational field<br>11= **ริมลำห้วยที่ผ่านไปการเกษตร** Agricultural streams<br>12 = **ริมลำห้วย หรือแม่น้ำในป่า** Rivers and streams in forest<br>13 = **ริม ถนน ทางเดิน หรือรั้ว** Roadside<br>14 = **อาหารที่แบ่งปันกัน ระบุ** Shared food, specify:<br>15 = **ที่อื่น ระบุ** Other place, specify: |

| | |
|---|---|
| **D6. เมื่อวานคุณได้รับประทานอาหาร ถั่วเหลือง, ถั่วเน่า, เต้าหู้, หรือดื่มนมถั่วเหลือง เช่น แลคตาซอย หรือไม่** Yesterday, did you eat any soybeans, tofu, or drink soymilk? | 0 = ไม่ใช่<br>1 = ใช่ |

| | |
|---|---|
| **D7. คุณได้ถั่วเหลืองที่กินเมื่อวานที่ไหน**<br>Where did you get the soy products that you ate or drank yesterday? | |

| **เมื่อวานคุณได้กินผักสีส้มเหล่านี้หรือไม่** Yesterday, did you eat any of the following vegetables: | |
|---|---|
| D8. แครอท, ฟักทอง, หรือมันหวานสีส้ม<br>Carrots, pumpkin, or sweet potatoes that are orange inside? | 0 = ไม่ใช่ 1 = ใช่ |

| **D8a. ผักสีส้มที่คุณกินเมื่อวาน ไปเก็บหรือซื้อ ได้มาจาก ไหน**<br>From which of the following types of places did you get the orange vegetables that you ate yesterday? | |
|---|---|
| 0 = **ไม่ได้กิน** Did not eat.<br>2= **ตลาดสด** Fresh market<br>2.5= **ร้านขายของชำ** Informal shop | 1= **ห้างสรรพสินค้า หรือร้านสะดวกซื้อ เช่น เทสโก้ โลตัส หรือเซเว่น**<br>Formal markets like Tesco Lotus or 7- Eleven<br>4 = **สวนทำการเกษตรเชิงพาณิชย์** Cash cropfield |

3 = **สวนผลไม้** Fruit orchard
5 = **สวนรอบบ้าน** Home garden
6 = **นาข้าว** Rice paddy
8 = **ไร่หมุนเวียน ที่กำลังปลูกอยู่** Planted rotational cropfield
10 = **ป่า** Forest
7 = **ไร่ / สวน** Subsistence crop field
9 = **ไร่หมุนเวียน ที่วางไว้** Fallow rotational field
11= **ริมลำห้วยที่ผ่านไปการเกษตร** Agricultural streams
12 = **ริมลำห้วย หรือแม่น้ำในป่า** Rivers and streams in forest
13 = **ริม ถนน ทางเดิน หรือรั้ว** Roadside
14 = **อาหารที่แบ่งปันกัน ระบุ** Shared food, specify:
15 = **ที่อื่น ระบุ** Other place, specify:

| **เมื่อวานคุณได้กินผักใบเขียวเหล่านี้หรือไม่** Yesterday, did you eat any of the following vegetables: | |
|---|---|
| D9. บล็อคโคลี่, ผักบุ้ง, คะน้า, ตำลึง, หรือผักใบเขียว (เช่นที่จิ้มน้ำพริก)<br>Broccoli, morning glory, Chinese kale, ivy gourd leaves, or other dark leaves? | 0 = ไม่ใช่ 1 = ใช่ |

**D9a. ผักใบเขียวที่คุณกินเมื่อวาน ไปเก็บหรือซื้อ ได้มาจาก ไหน**
From which of the following types of places did you get the vegetables that you ate yesterday?

0 = **ไม่ได้กิน** Did not eat.
2= **ตลาดสด** Fresh market
2.5= **ร้านขายของชำ** Informal shop
3 = **สวนผลไม้** Fruit orchard
5 = **สวนรอบบ้าน** Home garden
6 = **นาข้าว** Rice paddy
8 = **ไร่หมุนเวียน ที่กำลังปลูกอยู่** Planted rotational cropfield
10 = **ป่า** Forest
1= **ห้างสรรพสินค้า หรือร้านสะดวกซื้อ เช่น เทสโก้ โลตัส หรือเซเว่น** Formal markets like Tesco Lotus or 7- Eleven
4 = **สวนทำการเกษตรเชิงพาณิชย์** Cash cropfield
7 = **ไร่ / สวน** Subsistence crop field
9 = **ไร่หมุนเวียน ที่วางไว้** Fallow rotational field
11= **ริมลำห้วยที่ผ่านไปการเกษตร** Agricultural streams
12 = **ริมลำห้วย หรือแม่น้ำในป่า** Rivers and streams in forest
13 = **ริม ถนน ทางเดิน หรือรั้ว** Roadside
14 = **อาหารที่แบ่งปันกัน ระบุ** Shared food, specify:
15 = **ที่อื่น ระบุ** Other place, specify:

| **เมื่อวานคุณได้กินผักอื่น ๆ เหล่านี้หรือไม่** Yesterday, did you eat any of the following vegetables: | |
|---|---|
| D10. หน่อไม้, มะเขือยาว, ผักกะหล่ำ, ผักกาดหอม, ถั่วพู, ถั่วฝักยาว, หรือมะละกอดิบ<br>Bamboo shoots, eggplant, cabbage, lettuce, winged beans, long beans, or green papaya? | 0 = ไม่ใช่ 1 = ใช่ |
| D11. ฟักเขียว, บวบหอม หรือบวบเหลี่ยม, บวบน้ำเต้า, มะระ , ฟักแม้ว, แตงกวา, หรือมะเขือเทศ<br>Winter melon, sponge gourd or ridge gourd, bottle gourd, bitter gourd, chayote, cucumber, or tomatoes? | 0 = ไม่ใช่ 1 = ใช่ |
| D12. กะหล่ำดอก, เห็ด, หัวปลี, ดอกโสน, หรือถั่วงอก<br>Cauliflower, mushrooms, banana flower, Sesbania flower, or mung bean sprouts? | 0 = ไม่ใช่ 1 = ใช่ |

**D13. ผักอื่น ๆ ที่คุณกินเมื่อวาน ไปเก็บหรือซื้อ ได้มาจาก ไหน**
From which of the following types of places did you get the vegetables that you ate yesterday?

0 = **ไม่ได้กิน** Did not eat.
2= **ตลาดสด** Fresh market
2.5= **ร้านขายของชำ** Informal shop
3 = **สวนผลไม้** Fruit orchard
5 = **สวนรอบบ้าน** Home garden
6 = **นาข้าว** Rice paddy
8 = **ไร่หมุนเวียน ที่กำลังปลูกอยู่** Planted rotational cropfield
10 = **ป่า** Forest
1= **ห้างสรรพสินค้า หรือร้านสะดวกซื้อ เช่น เทสโก้ โลตัส หรือเซเว่น** Formal markets like Tesco Lotus or 7- Eleven
4 = **สวนทำการเกษตรเชิงพาณิชย์** Cash cropfield
7 = **ไร่ / สวน** Subsistence crop field
9 = **ไร่หมุนเวียน ที่วางไว้** Fallow rotational field
11= **ริมลำห้วยที่ผ่านไปการเกษตร** Agricultural streams
12 = **ริมลำห้วย หรือแม่น้ำในป่า** Rivers and streams in forest
13 = **ริม ถนน ทางเดิน หรือรั้ว** Roadside
14 = **อาหารที่แบ่งปันกัน ระบุ** Shared food, specify:
15 = **ที่อื่น ระบุ** Other place, specify:

| **เมื่อวานคุณได้กินผลไม้สีส้มเหล่านี้หรือไม่** Yesterday, did you eat any of the following orange fruit: | |
|---|---|
| D15. มะม่วงสุก, มะละกอสุก, เสาวรส, หรือแคนตาลูป<br>Ripe mango, ripe papaya, passion fruit, or canteloupe | 0 = ไม่ใช่ 1 = ใช่ |

**D15a. ผลไม้สีส้มที่คุณกินเมื่อวาน ไปเก็บหรือซื้อ ได้มาจาก ไหน**
From which of the following types of places did you get the vegetables that you ate yesterday?

0 = **ไม่ได้กิน** Did not eat.
2= **ตลาดสด** Fresh market
1= **ห้างสรรพสินค้า หรือร้านสะดวกซื้อ เช่น เทสโก้ โลตัส หรือเซเว่น** Formal markets like Tesco Lotus or 7- Eleven
4 = **สวนทำการเกษตรเชิงพาณิชย์** Cash cropfield

| | |
|---|---|
| 2.5= **ร้านขายของชำ** Informal shop<br>3 = **สวนผลไม้** Fruit orchard<br>5 = **สวนรอบบ้าน** Home garden<br>6 = **นาข้าว** Rice paddy<br>8 = **ไร่หมุนเวียน ที่กำลังปลูกอยู่** Planted rotational cropfield<br>10 = **ป่า** Forest | 7 = **ไร่ / สวน** Subsistence crop field<br>9 = **ไร่หมุนเวียน ที่วางไว้** Fallow rotational field<br>11= **ริมลำห้วยที่ผ่านไปการเกษตร** Agricultural streams<br>12 = **ริมลำห้วย หรือแม่น้ำในป่า** Rivers and streams in forest<br>13 = **ริม ถนน ทางเดิน หรือรั้ว** Roadside<br>14 = **อาหารที่แบ่งปันกัน ระบุ** Shared food, specify:<br>15 = **ที่อื่น ระบุ** Other place, specify: |

| **เมื่อวานคุณได้กินผลไม้อื่น ๆ เหล่านี้หรือไม่** Yesterday, did you eat any of the following fruit: | |
|---|---|
| D16. ส้มหรือส้มโอ<br>Oranges or pomelo | 0 = ไม่ใช่ 1 = ใช่ |
| D17. กล้วย, แตงโม, เมลอน, แอปเปิล, ฝรั่ง, น้อยหน่า, หรือสัปปะรด<br>Banana, watermelon, melon, apple, guava, custard apple, or pineapple? | 0 = ไม่ใช่ 1 = ใช่ |
| D18. ทุเรียน, มังคุด, ขนุน, แก้วมังกร, มะพร้าวอ่อน, เงาะ, ลางสาด, หรือลำไย<br>Durian, mangosteen, jackfruit, dragonfruit, young coconut, rambutan, langsat, or longan? | 0 = ไม่ใช่ 1 = ใช่ |
| D19. มะม่วงดิบ,สาลี่, ชมพู่, องุ่น,พุทราจีน, มะเฟือง หรือสตรอเบอร์รี่<br>Green mango, Chinese pear, rose apple, grapes, jujube, star fruit, or strawberries? | 0 = ไม่ใช่ 1 = ใช่ |

| **D20. ผลไม้ที่คุณกินเมื่อวาน ได้มาจาก ไหน**<br>From which of the following types of places did you get the fruits that you ate yesterday? | |
|---|---|
| 0 = **ไม่ได้กิน** Did not eat.<br>2= **ตลาดสด** Fresh market<br>2.5= **ร้านขายของชำ** Informal shop<br>3 = **สวนผลไม้** Fruit orchard<br>5 = **สวนรอบบ้าน** Home garden<br>6 = **นาข้าว** Rice paddy<br>8 = **ไร่หมุนเวียน ที่กำลังปลูกอยู่** Planted rotational cropfield<br>10 = **ป่า** Forest | 1= **ห้างสรรพสินค้า หรือร้านสะดวกซื้อ เช่น เทสโก้ โลตัส หรือเซเว่น** Formal markets like Tesco Lotus or 7- Eleven<br>4 = **สวนทำการเกษตรเชิงพาณิชย์** Cash cropfield<br>7 = **ไร่ / สวน** Subsistence crop field<br>9 = **ไร่หมุนเวียน ที่วางไว้** Fallow rotational field<br>11= **ริมลำห้วยที่ผ่านไปการเกษตร** Agricultural streams<br>12 = **ริมลำห้วย หรือแม่น้ำในป่า** Rivers and streams in forest<br>13 = **ริม ถนน ทางเดิน หรือรั้ว** Roadside<br>14 = **อาหารที่แบ่งปันกัน ระบุ** Shared food, specify:<br>15 = **ที่อื่น ระบุ** Other place, specify: |

| **เมื่อวานคุณได้กินของหวานเหล่านี้หรือไม่**<br>Yesterday, did you eat any of the following sweets: | |
|---|---|
| D21. เค้ก, คุกกี้, โดนัท, หรือของหวานเบเกอรี่<br>Cakes, cookies, donuts, or other sweet bakery products? | 0 = ไม่ใช่<br>1 = ใช่ |
| D22. ลูกอม, ช็อคโกแลต, ไอศกรีม, พุดดิ้ง, ขนมหวานจากกระทิ, ข้าวเหนียวหน้าต่างๆ หรือข้าวเหนียวกับผลไม้, หรือขนมไทยต่างๆ<br>Candies, chocolates, ice cream, pudding, coconut milk dessert, sticky rice with topping or with fruit, or other Thai dessert? | 0 = ไม่ใช่<br>1 = ใช่ |

| | |
|---|---|
| **D23. ขนมที่คุณกินเมื่อวาน ได้มาจาก ไหน**<br>From which of the following types of places did you get the sweets that you ate yesterday? | 0 = **ไม่ได้กิน**<br>1= **ห้างสรรพสินค้า หรือร้านสะดวกซื้อ เช่น เทสโก้ โลตัส หรือเซเว่น** Formal markets like Tesco Lotus or 7- Eleven<br>2= **ตลาดสด** Fresh market 2.5= **ร้านขายของชำ** Informal shop<br>14 = **อาหารที่แบ่งปันกัน** Shared food<br>15 = **ที่อื่น ระบุ** Other place (specify):_______________________ 99 = **ไม่รู้** |

| **เมื่อวานคุณได้กินที่มีวัตถุดิบจากสัตว์เหล่านี้หรือไม่** Yesterday, did you eat any of the following foods of animal origin: | |
|---|---|
| D24. ไข่ Eggs? | 0 = ไม่ใช่ 1 = ใช่ |
| D25. ชีส Cheese? | 0 = ไม่ใช่ 1 = ใช่ |
| D26. โยเกิร์ต หรือนมเปรี้ยว Yoghurt or sour milk? | 0 = ไม่ใช่ 1 = ใช่ |

| | |
|---|---|
| **D27. ไข่ ชีส โยเกิร์ต หรือนมเปรี้ยว ที่คุณได้กินเมื่อวาน ได้ที่ไหน**<br><br>Where did you get the eggs, cheese, yoghurt or sour milk that you ate yesterday? | 0 = ไม่ได้กิน<br>1= **ห้างสรรพสินค้า หรือร้านสะดวกซื้อ เช่น เทสโก้ โลตัส หรือเซเว่น** Formal markets like Tesco Lotus or 7- Eleven<br>2= **ตลาดสด** Fresh market 2.5= **ร้านขายของชำ** Informal shop<br>3 = **คนขาย และเลี้ยงในหมู่บ้าน** Animal sources in village<br>4 = **สัตว์ของคุณ เช่น ไข่ไก่อยู่ใกล้บ้าน** Own animals<br>5 = **ที่อื่น ระบุ** Other place (specify):________________________ 9 = **ไม่รู้** |

| **เมื่อวานคุณได้รับประทานอาหารที่มีวัตถุดิบจากสัตว์เหล่านี้หรือไม่**<br>Yesterday, did you eat any of the following foods of animal origin: | |
|---|---|
| D28. ไส้กรอก,เบคอน,เนื้อสัตว์ตาก แห้ง,แฮมหรือโบโลญญา<br>Sausages, bacon, dried meat, ham, or bologna? | 0 = ไม่ใช่ 1 = ใช่ |
| D29. เนื้อวัว, เนื้อแพะ, หรือเนื้อควาย Beef, goat, or buffalo meat? | 0 = ไม่ใช่ 1 = ใช่ |
| D30. เนื้อหมู, กบ, กระต่าย, หนูนา, เลือดหมู, หรือเครื่องในหม<br>Pork, pork liver, frog, rabbit, field mouse or pig blood? | 0 = ไม่ใช่ 1 = ใช่ |
| D31. ไก่, เป็ด , ห่าน, นกป่า, หรือเลือดนก Chicken, duck, goose, wild birds, or chicken blood? | 0 = ไม่ใช่ 1 = ใช่ |

**D32. เนื้อสัตว์ ที่คุณกินเมื่อวาน ได้ซื้อ จับ หรือล่า ได้มาจาก ไหน**
From which of the following types of places did you get the meat that you ate yesterday?

| | |
|---|---|
| 0 = **ไม่ได้กิน** Did not eat.<br>2= **ตลาดสด** Fresh market<br>2.5= **ร้านขายของชำ** Informal shop<br>3 = **สวนผลไม้** Fruit orchard<br>5 = **สวนรอบบ้าน** Home garden<br>6 = **นาข้าว** Rice paddy<br>8 = **ไร่หมุนเวียน ที่กำลังปลูกอยู่** Planted rotational cropfield<br>10 = **ป่า** Forest | 1= **ห้างสรรพสินค้า หรือร้านสะดวกซื้อ เช่น เทสโก้ โลตัส หรือเซเว่น** Formal markets like Tesco Lotus or 7- Eleven<br>4 = **สวนทำการเกษตรเชิงพาณิชย์** Cash cropfield<br>7 = **ไร่ / สวน** Subsistence crop field<br>9 = **ไร่หมุนเวียน ที่วางไว้** Fallow rotational field<br>15 = **คนขาย และเลี้ยงในหมู่บ้าน** Butcher/livestock owner in village<br>16 = **สัตว์ของคุณ เช่น ไข่ไก่อยู่ใกล้บ้าน** Own animals<br>17 = **ที่อื่น ระบุ** Other place (specify):______________________________<br>99 = **ไม่รู้** |

| | |
|---|---|
| **D33. เมื่อวานคุณได้รับประทานอาหาร ปลา ปลาตัวเล็ก ปลาหมัก อาหารทะเล กุ้ง ปู หรือหอยไหม**<br>Yesterday did you eat fish, small fish, fermented fish, seafood, shrimp, prawn, crabs or molluscs? | 0 = ไม่ใช่<br>1 = ใช่ |

**D34. ปลา, ปลาหมัก, อาหารทะเล, กุ้ง, ปู, หรือหอย ที่คุณได้กินเมื่อวาน ได้มาจาก ไหน**
From which of the following types of places did you get the fish and seafood that you ate yesterday?

| | |
|---|---|
| 0 = **ไม่ได้กิน** Did not eat.<br>2= **ตลาดสด** Fresh market<br>2.5= **ร้านขายของชำ** Informal shop<br>3 = **สวนผลไม้** Fruit orchard<br>5 = **สวนรอบบ้าน** Home garden<br>6 = **นาข้าว** Rice paddy<br>8 = **ไร่หมุนเวียน ที่กำลังปลูกอยู่** Planted rotational cropfield<br>10 = **ป่า** Forest | 1= **ห้างสรรพสินค้า หรือร้านสะดวกซื้อ เช่น เทสโก้ โลตัส หรือเซเว่น** Formal markets like Tesco Lotus or 7- Eleven<br>4 = **สวนทำการเกษตรเชิงพาณิชย์** Cash cropfield<br>7 = **ไร่ / สวน** Subsistence crop field<br>9 = **ไร่หมุนเวียน ที่วางไว้** Fallow rotational field<br>11= **ริมลำห้วยที่ผ่านไปการเกษตร** Agricultural streams<br>12 = **ริมลำห้วย หรือแม่น้ำในป่า** Rivers and streams in forest<br>13 = **บ่อน้ำหรือทะเลสาบ** Roadside Pond or lake<br>14 = **อาหารที่แบ่งปันกัน ระบุ** Shared food, specify:<br>15 = **ที่อื่น ระบุ** Other place, specify: |

| | |
|---|---|
| **D35. เมื่อวานคุณได้รับประทานอาหารถั่วลิสง เม็ดมะม่วงหิมพานต์ เมล็ดทานตะวัน เมล็ดฟักทอง หรือเมล็ดแตงโม หรือไม่** Yesterday did you eat any peanuts, cashews, sunflower seeds, pumpkin seeds, or watermelon seeds? | 0 = ไม่ใช่<br>1 = ใช่ |

**D36. ถั่วลิสง เม็ดมะม่วงหิมพานต์ และเมล็ด ที่คุณกินเมื่อวาน ได้มาจากไหน**
From which of the following types of places did you get the nuts and seeds that you ate yesterday?

| | |
|---|---|
| 0 = **ไม่ได้กิน** Did not eat.<br>2= **ตลาดสด** Fresh market<br>2.5= **ร้านขายของชำ** Informal shop<br>3 = **สวนผลไม้** Fruit orchard | 1= **ห้างสรรพสินค้า หรือร้านสะดวกซื้อ เช่น เทสโก้ โลตัส หรือเซเว่น** Formal markets like Tesco Lotus or 7- Eleven<br>4 = **สวนทำการเกษตรเชิงพาณิชย์** Cash cropfield<br>7 = **ไร่ / สวน** Subsistence crop field |

| | |
|---|---|
| 5 = **สวนรอบบ้าน** Home garden<br>6 = **นาข้าว** Rice paddy<br>8 = **ไร่หมุนเวียน ที่กำลังปลูกอยู่** Planted rotational cropfield<br>10 = **ป่า** Forest | 9 = **ไร่หมุนเวียน ที่วางไว้** Fallow rotational field<br>11= **ริมลำห้วยที่ผ่านไปการเกษตร** Agricultural streams<br>13 = **ริม ถนน ทางเดิน หรือรั้ว** Roadside<br>14 = **อาหารที่แบ่งปันกัน ระบุ** Shared food, specify:<br>15 = **ที่อื่น ระบุ** Other place, specify: |

| **เมื่อวานคุณได้รับประทานอาหาร อาหารเหล่านี้หหรือไม่** Yesterday, did you eat any of the following other foods: | |
|---|---|
| D37. มันฝรั่งแผ่นทอด, ข้าวเกรียบกุ้ง, หรือขนมกรุบกรอบ<br>Potato chips, shrimp chips, or other kanom krup krob? | 0 = ไม่ใช่<br>1 = ใช่ |
| D38. บะหมี่กึ่งสำเร็จรูป, โจ๊กคัพ, หรือซุปพร้อมทาน<br>Instant noodles, cup of porridge, or instant soup? | 0 = ไม่ใช่<br>1 = ใช่ |
| D39. มันฝรั่งทอด, มันหวานทอด, กล้วยทอด, ปอเปี๊ยะ, เกี๊ยวทอด, ไก่ทอด, ลูกชิ้นปลาทอด หรือลูกชิ้นทอด<br>Fried potato, fried sweet potato, fried banana, spring rolls, fried wontons, fried fish balls, or fried meat balls? | 0 = ไม่ใช่<br>1 = ใช่ |

| | |
|---|---|
| **D40. ขนมกรุบกรอบ ขนมทอด หรือบะหมี่มาม่า ที่คุณได้กิน เมื่อวาน ได้มาจาก ไหน**<br>Where did you get the crunchy and fried snacks and instant noodles that you ate yesterday? | 0 = **ไม่ได้กิน**<br>1= **ห้างสรรพสินค้า หรือร้านสะดวกซื้อ เช่น เทสโก้ โลตัส หรือเซเว่น** Formal markets like Tesco Lotus or 7- Eleven<br>2= **ตลาดสด** Fresh market 2.5= **ร้านขายของชำ** Informal shop<br>14 = **อาหารที่แบ่งปันกัน ระบุ** Shared food, specify:<br>15 = **ที่อื่น ระบุ** Other place, specify:__________________________ 99 = **ไม่รู้** |

| **เมื่อวานคุณดื่มเครื่องดื่มเหล่านี้ หรือไม่** Yesterday, did you drink any of the following beverages: | |
|---|---|
| D41. นมวัว, นมแพะ, หรือนมผง<br>Cow milk, goat milk, or powdered milk? | 0 = ไม่ใช่<br>1 = ใช่ |
| D42. กาแฟหวาน, ชาหวาน, ชาขวดหวาน, ชาไข่มุก, นมรสชาติต่างๆ, หรือเครื่องดื่มช็อคโกแลต หรือโกโก<br>Sweetened coffee, sweetened tea, bottled sweet tea, flavored milk, or chocolate or cacao drink? | 0 = ไม่ใช่<br>1 = ใช่ |
| D43. น้ำผลไม้สด, น้ำผลไม้กล่อง, น้ำผลไม้ปั่น, สมูทตี้, น้ำกระเจี๊ยบ, น้ำใบเตย, หรือน้ำสมุนไพรอื่นๆ<br>Fruit juice, fruit drinks, fruit shake, smoothie, roselle juice, pandan juice, or other herbal drinks? | 0 = ไม่ใช่<br>1 = ใช่ |
| D44. น้ำอัดลม เช่น เป๊ปซี่, โคล่า, เอส, เครื่องดื่มชูกำลัง เช่น กระทิงแดง, เอ็มร้อยห้าสิบ, หรือเกเตอเรท<br>Soda such as Pepsi, Coca-Cola, or energy drinks such as Red Bull or M-150, or Gatorade? | 0 = ไม่ใช่<br>1 = ใช่ |

| **D45. คุณได้เครื่องดื่มที่ดื่มเมื่อวานที่ไหน** Where did you get the beverages that you drank yesterday? | |
|---|---|
| 0 = **ไม่ได้กิน** Did not eat.<br>2= **ตลาดสด** Fresh market<br>2.5= **ร้านขายของชำ** Informal shop<br>3 = **สวนผลไม้** Fruit orchard<br>5 = **สวนรอบบ้าน** Home garden<br>6 = **นาข้าว** Rice paddy<br>8 = **ไร่หมุนเวียน ที่กำลังปลูกอยู่** Planted rotational cropfield<br>10 = **ป่า** Forest | 1= **ห้างสรรพสินค้า หรือร้านสะดวกซื้อ เช่น เทสโก้ โลตัส หรือเซเว่น** Formal markets like Tesco Lotus or 7- Eleven<br>4 = **สวนทำการเกษตรเชิงพาณิชย์** Cash cropfield<br>7 = **ไร่ / สวน** Subsistence crop field<br>9 = **ไร่หมุนเวียน ที่วางไว้** Fallow rotational field<br>11= **ริมลำห้วยที่ผ่านไปการเกษตร** Agricultural streams<br>13 = **ริม ถนน ทางเดิน หรือรั้ว** Roadside<br>14 = **อาหารที่แบ่งปันกัน ระบุ** Shared food, specify:<br>15 = **ที่อื่น ระบุ** Other place, specify: |

| | |
|---|---|
| **D46. เมื่อวานคุณได้รับประทานจากสถานที่เหล่านี้หรือไม่ เคเอฟซี, แมคโดนัล, พิซซ่า, เชสเตอร์กริล, หรือสถานที่อื่นๆ ที่เสิร์ฟเบอร์เกอร์, ไก่ทอดหรือพิซซ่า** Yesterday, did you get food from any place like KFC, McDonald's, Pizza Company, Chester Grill or other places that serve burgers, fried chicken or pizza? | 0 = ไม่ใช่<br>1 = ใช่ |

| **D47. เมื่อวานคุณกินหรือดื่มอะไรอีกที่ไม่รวมในคำถามเหล่านี้ไหม** Yesterday, did you eat or drink anything else? |
|---|
| |
