# Supplementary material for: Adapting food environment frameworks to recognize a wild-cultivated continuum
Source: Front Nutr. 2024 Apr 9;11:1343021. doi: 10.3389/fnut.2024.1343021 (PMC11035871; doi:10.3389/fnut.2024.1343021)
Supplement: Supplementary file 2 [file Table_1_1.pdf]

Supplementary Information

Table 1. *Numbers of food items reported for each food group by type of food environment in San Din Daeng village, using the Gallup Poll's Thailand-adapted Diet Quality Questionnaire (n=31).*

	Formal Market	Informal Market	Wild-Cultivated	Cultivated	Wild	Food Shared from Unknown	Food Shared from Wild-Cultivated	Food Shared from Informal Market	Unknown Source	Total Number of Food Items
Grains	0	6 (16%)	30 (79%)	0	0	0	1 (3%)	1 (3%)	0	38
Tubers	0	1 (33%)	0	0	0	1 (33%)	1 (33%)	0	0	3
Pulses	0	8 (80%)	1 (10%)	0	0	0	0	1 (10%)	0	10
Vitamin A-rich vegetables	0	0	7 (78%)	0	0	0	1 (11%)	0	1 (11%)	9
Dark green leafy vegetables	0	3 (18%)	10 (59%)	0	0	0	1 (6%)	1 (6%)	2 (12%)	17
Other vegetables	0	16 (24%)	21 (31%)	0	13 (19%)	3 (4%)	5 (7%)	2 (3%)	7 (10%)	67
Vitamin A-rich fruits	0	0	1 (50%)	0	0	0	1 (50%)	0	0	2
Other fruits	0	5 (18%)	15 (54%)	0	0	3 (11%)	4 (14%)	1 (4%)	0	28
Eggs	0	15 (100%)	0	0	0	0	0	0	0	15
Dairy	0	4 (100%)	0	0	0	0	0	0	0	4
Meat	0	15 (56%)	3 (11%)	0	3 (11%)	4 (15%)	0	2 (7%)	0	27
Fish	0	13 (72%)	0	0	2 (11%)	1 (6%)	0	2 (11%)	0	18
Nuts and Seeds	0	1 (50%)	0	0	0	1 (50%)	0	0	0	2
Total Number of Food Items	*0*	*87*	*88*	*0*	*18*	*13*	*14*	*10*	*10*	*240*
Average Percentage	*0*	*36*	*37*	*0*	*8*	*5*	*6*	*4*	*4*	
